# Point mutation in *GRIM-19*: a new genetic lesion in Hurthle cell thyroid carcinomas

**DOI:** 10.1038/sj.bjc.6602556

**Published:** 2005-05-17

**Authors:** A Fusco, G Viglietto, M Santoro

**Affiliations:** 1 Dipartimento di Biologia e Patologia Cellulare e Molecolare c/o Istituto di Endocrinologia ed Oncologia Sperimentale del CNR, Facolta di Medicina e Chirurgia di Napoli, Universita degli Studi di Napoli ‘Federico M’, via Pansini, 5, 80131 Naples, Italy; 2 NOGEC (Naples Oncogenomic Center)-CEINGE, Biotecnologie Avanzate, via Comunale Margherita 482, 80145 Naples, Italy; 3 Dipartimento di Medicina Sperimentale e Clinica, Universita degli Studi di Catanzaro ‘Magna Graecia’, viale Europa, 88100 Catanzaro, Italy

A very peculiar group of tumours of the thyroid gland is characterised by the presence of large polygonal cells with a distinctive granular eosinophilic cytoplasm packed with mitochondria. This type of cell has been referred to as oncocyte, Hurthle, Askanazy or oxyphil cell (Sobrinho-Simoes *et al*, 2004). Hurthle cell tumours (HCT) are defined as being composed of at least 75% Hurthle cells. Hurthle cell adenomas (HCA) are encapsulated benign lesions, while Hurthle cell carcinomas (HCC) can be classified as variant of follicular thyroid carcinoma (FTC) or papillary thyroid carcinoma (PTC) (Sobrinho-Simoes *et al*, 2004).

Molecular bases of the vast majority of HCT are still largely unknown. Hurthle cell carcinomas variant of PTC is characterised by RET/PTC rearrangements and BRAF^V800E^ mutations, hallmarks of classic PTC cases. Hurthle cell carcinomas often show aneuploid karyotype with widespread numerical chromosomal alterations and frequent chromosome 7 trisomy (Dettori *et al*, 2003). Moreover, allelic losses at 19p13.2 and 2q21 are prevalent in HCT. In these regions, two loci *(TOO,* thyroid tumours with cell oxyphilia, and *NMTC1,* nonmedullary thyroid carcinoma 1) predisposing to familial nonmedulllary thyroid carcinoma (FNMTC) have been mapped (Stankov *et al*, 2004). However, the corresponding genes have not been isolated yet.

Mitochondria have been proposed to play an important role in HCT formation. The increased number of mitochondria and the mitochondrial structural abnormalities observed in HCT mimic those detected in the cells of patients with several mitochondrial diseases and myopathies. Mitochondria play essential roles in cellular energy production and it has been proposed that mitochondrial proliferation in HCT might be a compensatory mechanism for a decline in oxidativephosphorylation (Maximo *et al*, 2002). The NADH:ubiquinone oxidoreductase (complex I) catalyses the first step of electron transfer in the mitochondrial oxidative phosphorylation system and it is encoded by nuclear and mithocondrial genes. Mitochondrial DNA (mtDNA) mutations are often present in HCT and most of them target genes that belong to the complex I (Maximo *et al*, 2002). The high rate of mtDNA replication coupled with the inherent instability of mtDNA have been suggested to cause such a high rate of mtDNA mutations. Moreover, genome-wide expression profiling has revealed imbalance in the expression of mitochondrial genes and in nuclear genes encoding the respiratory chain complexes in HCT.

In this issue of BJC, Maximo and co-workers describe a novel genetic lesion in HCC of the thyroid (Maximo *et al*, 2005, present issue). Their discovery highlights a novel intriguing connection at the genetic level between HCT occurrence, mitochondrial metabolism and cell death. In particular, Maximo *et al* described HCC cases carrying mutations in the *GRIM-19* gene. *GRIM-19* (gene associated with retinoid-interferon-induced mortality-19) maps on 19p13.2 and codes for a 16-kDa protein that may be localised in the nucleus and mithocondria. The GRIM-19 protein exerts a dual function: (i) it is essential for assembly and function of the complex I of the mitochondrial respiratory chain (Huang *et al*, 2004) and (ii) it induces apoptosis in a number of cell lines upon treatment with interferon-beta and retinoic acid (Angell *et al*, 2000). Mechanistically, this could be, at least in part, linked to the capability of the GRIM-19 protein to bind to and suppress transcription driven by STATS (signal transducer and activator of transcription 3) (Lufei *et al*, 2003). STATS plays important roles in cell growth, survival and cell transformation, and is constitutively active in various cancers. Moreover, GRIM-19 interacts with a protein named GW112 that is highly expressed in colon cancers and has an antiapoptotic function (Zhang *et al*, 2004). Mutations identified in HCC by Maximo *et al* were located at codons 26, 83, 88 (exon 1) and 198 (exon 5) of *GRIM-19.* In one of the cases (case 7) there was a papillary carcinoma, displaying Hurthle cell features in which there was a RET/PTC rearrangement in addition to the GRIM 19 mutation. Three of the GRIM-19 mutations were somatic, while the other one (codon 88) was germline. In the latter case there were, besides the Hurthle cell variant of PTC, multiple benign nodules displaying Hurthle cell features. However, no GRIM-19 mutations have been found in six families affected by familial HTC, even though GRIM-19 maps on chromosome 19p13.2 where also the TCO locus has been located.

There is good reason to believe that the report by Maximo *et al* will represent an important step forward in our molecular understanding of HCT and of mitochondrial disfunction in this tumour type. The mitochondrial and the cell survival activity of *GRIM-19,* coupled with the presence of abnormal mitochondrial structures in HCT, suggest that, indeed, *GRIM-19* mutations may be important for HCT pathogenesis. However, it will be crucial to determine the functional consequences of such mutations. Since *GRIM-19* is a proapoptotic gene, it is tempting to speculate that its loss-of-fucntion may contribute to HCC. The mutations identified by Maximo and co-workers were not associated to loss of heterozygosity; therefore, an important point to be addressed is whether they act through haploinsufficiency or negative dominance mechanisms. Ablation of *GRIM-19* expression by RNA interference in cultured thyrocytes and thyroid specific-targeting in transgenic mice could provide formal proofs of *GRIM-19* role in thyroid carcinogenesis. Finally, the analysis of larger series of samples could confirm the prevalence of *GRIM-19* mutations and their distribution in the various HCT subtypes. It will be also interesting to explore whether *GRIM-19* is involved in oxyphilic tumours affecting nonthyroid tissues such as kidney, salivary and parathyroid glands tumours. Finally, the results by Maximo and co-workers will prompt the search for mutations in other genes with similar functions in human tumours.
